# Spectral Characterization of Stem Cell-Derived Myelination within the Injured Adult PNS Using the Solvatochromic Dye Nile Red

**DOI:** 10.3390/cells9010189

**Published:** 2020-01-11

**Authors:** Joey Grochmal, Wulin Teo, Hardeep Gambhir, Ranjan Kumar, Jo Anne Stratton, Raveena Dhaliwal, Craig Brideau, Jeff Biernaskie, Peter Stys, Rajiv Midha

**Affiliations:** 1 Division of Neurosurgery, Department of Surgery, Texas Tech University, Lubbock, TX 79415, USA; 2 Hotchkiss Brain Institute, Calgary, AB T2N 4N1, Canada; wulin.teo@gmail.com (W.T.); hgambhir@gmail.com (H.G.); rkum@ucalgary.ca (R.K.); cbrideau@ucalgary.ca (C.B.); jeff.biernaskie@ucalgary.ca (J.B.); pstys@ucalgary.ca (P.S.); Rajiv.Midha@albertahealthservices.ca (R.M.); 3 Department of Clinical Neurosciences, Cumming School of Medicine, University of Calgary, Calgary, AB T2N 4N1, Canada; raveena.d19@gmail.com; 4 Department of Comparative Biology and Experimental Medicine, University of Calgary, Calgary, AB T2N 4N1, Canada; 5 The Neuro, Montreal Neurological Institute, 3801 University Drive, Montreal, QC H3A 2B4, Canada; jo.stratton@mcgill.ca

**Keywords:** intravital imaging, myelin, PNS, remyelination, spectral confocal microscopy, solvatochromism

## Abstract

Background: Myelin is an essential component of the peripheral and central nervous system, enabling fast axonal conduction and supporting axonal integrity; limited tools exist for analysis of myelin composition in-vivo. Objective: To demonstrate that the photophysical properties of myelin-incorporated solvatochromic dyes can be exploited to probe the biochemical composition of living peripheral nerve myelin at high spatial resolution. Methods: Using the myelin-incorporated fluorescent dye Nile Red we sequentially analyzed the spectral characteristics of remyelinating myelin membranes both in-vitro and in-vivo, including in living rats. Results: We demonstrated a consistent bi-phasic evolution of emission spectra during early remyelination, and visually report the reliable biochemical flux of myelin membrane composition in-vitro and in-vivo. Conclusions: Solvatochromic spectroscopy enables the analysis of myelin membrane maturity during remyelination, and can be performed in-vivo. As the formation of myelin during early-to-late remyelination likely incorporates fluctuating fractions of lipophilic components and changes in lateral membrane mobility, we propose that our spectrochemical data reflects the observation of these biochemical processes.

## 1. Introduction

A rapidly expanding field of biophysical science is the analysis of biological membranes by environmentally sensitive fluorescent probes [1]. Solvatochromic fluorophores are one such class of probe. Upon excitation, many solvatochromic compounds (such as Nile Red) attain a large intramolecular dipole [2]. The reaction of the surrounding molecular environment (or “solvent sphere”) to the dipole is a near-instant, stability-inducing interaction that decreases the energy of the excited state. A mobile and polar solvent will provide maximal stability to the excited fluorophore, and hence result in low energy (red-shifted) emission wavelengths. Highly ordered and non-polar environments result in less stabilization and a higher energy, blue-shifted emission [3,4,5,6,7,8].

In this study we apply the concepts of solvatochromism to the study of the complex biological process of remyelination in the peripheral nervous system. We show that spectral analysis of Nile Red emission can be used to study the dynamics of regenerating myelin biochemistry, with important applications for the study of living tissue and live animals. Our results indicate that the regenerating myelin membrane undergoes a biphasic progression in biochemical composition from early to late remyelination, a process which evolves over approximately two weeks. As the observed spectral changes are a mirror to the biochemical evolution of the myelin lipid environment, the ability to image this process at high resolution is a considerable advancement for laboratory studies concerned with myelin health in the peripheral nervous system, and may have potential application for use in clinical scenarios.

## 2. Methods

### 2.1. Spectral Imaging and Data

Spectral imaging improves upon standard 3 or 4-channel confocal analysis by instead using up to 32 channels, in order to sample a large portion of the visible light spectrum at regular intervals. This sampling allows the construction of a wavelength vs. intensity curve that is a more detailed representation of the fluorescent characteristics of a sample of interest. In our experiments, 32 channels (separated by 10 nm) recorded fluorescence intensity for every pixel of the captured image/data set, spanning 320 nm of the visible light spectrum. The intensity vs wavelength curve generated for each pixel was the basic unit of spectral data used for our experimental analysis. In particular, when an individual myelin sheath was manually highlighted in our analysis (as all of our analysis was done), it contained numerous pixels that each contributed their basic spectral data to characterize the region of interest.

Using these fundamental pixel-specific data, we have recently established a novel method for the quantification of spectral data [9]. We found this necessary, as standard analysis of wavelength vs. intensity curves are highly skewed towards reporting the spectra of the brightest pixels in a particular selection region, which tends to account for only a minority of the total area of myelin analyzed [9].

**Figure 1:**
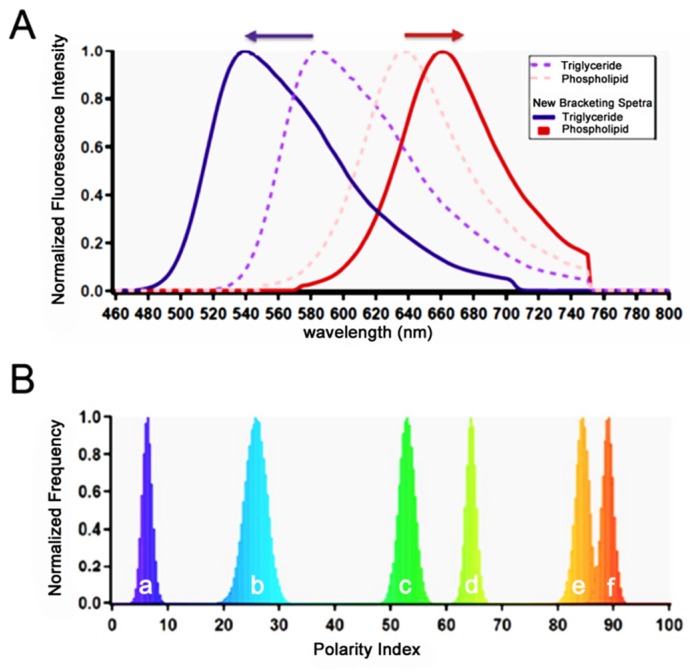
Polarity index measurements by Nile Red spectral shifts. (**A**) The blue edge bracketing spectra (solid blue line, 540 nm peak) is derived from the Invitrogen library, using the spectra of Nile Red emission as dissolved in triglyceride (553 nm peak, broken purple line), and shifting it to the blue by 13 nm. In a similar fashion, the red edge bracketing spectra (solid red line) is also derived from the Invitrogen library (Nile Red emission as dissolved in phospholipid, 637 nm peak, broken pink line) by shifting it to the red by 23 nm. (**B**) Emission histogram data for monomolecular solvents expressed as Polarity Index: (a) hexane, (b) Toluene, (c) chloroform (d) acetone (e) DMSO (f) Methanol. In this fashion, the x-axis covers enough emission breadth to capture emission data from Nile Red in opposite extremes of monomolecular polarity.

Instead we use a new metric termed the “polarity index” [9], which characterizes every pixel’s spectrum into a histogram data set that demonstrates the most common spectra to be found in a region of interest. The polarity index value is expressed as a percentage; it is a measurement of where the spectral peak wavelength values from individual pixels (the wavelength at which intensity is a maximum) in a selection region fall between two bracketing spectra. Values close to 0% imply that a pixel’s spectrum is close to the blue-shifted bracketing reference spectrum, while indexes approaching 100% reflect spectra close to the red-shifted reference spectrum. This allowed the determination of not only the most frequently measured polarity index in a selection region (PI, a measure of central tendency of polarity) but also of the width of the population as a whole; these measures were more sensitive than an overall average. For bracketing spectra, standardized reference spectra were obtained for Nile Red emission in highly polar (phospholipid) and non-polar (triglyceride) solvents from the Thermofisher Spectra-viewer webpage (thermofisher.com/order/spectra-viewer, Thermofisher, Waltham, MA, USA). These bracketing spectra were then further shifted horizontally to their respective blue and red max positions by 13 nm, in order to encompass the entire scope of potential Nile red emission in monomolecular solvents, and enabling a breadth of analysis capable of capturing any Nile Red emission in biological membranes (Figure 1). As this scale encompasses all potential Nile Red emission, pixel specific spectral data will fall between the two extremes of the bracketing spectra, and are assigned a percentage value based on their similarity to either bracket. Multiple pixel selection regions give histogram style data, that are distributed around the most frequent Polarity Index value demonstrated by those pixels. We report this peak/modal x-axis value (where the normalized frequency = 1.0) as the overall polarity index (PI) for a selection region, providing us with a means of analyzing large regions independent of the variations in fluorescence intensity within a particular selection.

### 2.2. Spectral Image Processing

All images were captured as 32 channel spectral data sets spanning 320 nm, using a spectral detector [multi-anode photomultiplier tube (Hamamatsu, Bridgewater, NJ, USA)]. Images were saved within the Nikon EzC1 software (Nikon, Mississauga, ON, Canada), then analyzed using ImageTrak (written by PKS, v4.12.13, https://www.ucalgary.ca/styslab/imagetrak, Calgary, AB, Canada).

### 2.3. Cell Culture and Histology

Culture and transfection of BFP-SKP-SCs and Schwann cells, as well as harvest, culture, and respective co-culture of Thy-1 GFP DRG explants, along with relevant histological processing were performed according to established methods [10].

### 2.4. Experimental Designs for Analysis of Remyelination Spectra

**Figure 2:**
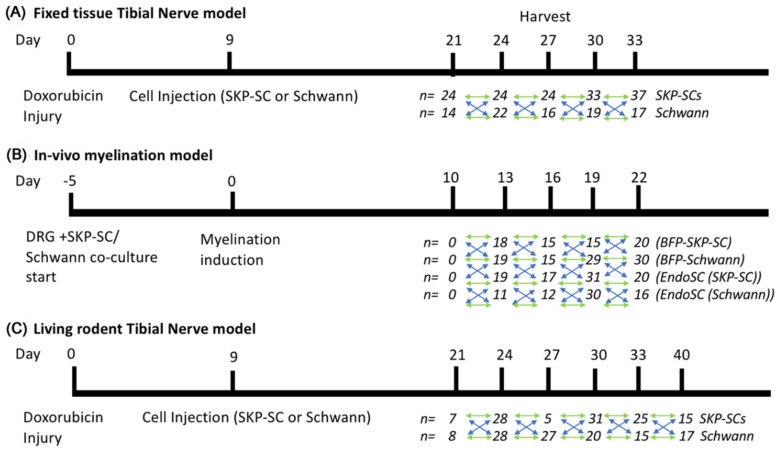
Experimental timelines for each experimental paradigm of exogenous or endogenous cell-derived myelination. (**A**) Timeline for fixed tissue analysis of injected skin-derived precursor Schwann cell (SKP-SC) and Schwann cell myelin within a tibial nerve doxorubicin injury model. (**B**) Timeline for an analysis of in vitro myelination provided by blue fluorescent protein skin-derived precursor Schwann cells (BFP SKP-SCs) and endogenous green fluorescent protein (GFP) Schwann cells (EndoSC (SKP-SC)) in co-culture with GFP-positive dorsal root ganglia (DRG) explants, alongside an analysis of BFP-Schwann cells and endogenous GFP Schwann cells (EndoSC (Schwann)) likewise in co-culture with GFP DRG explants. (**C**) Timeline for the analysis of living myelin by BFP-SKP-SCs and BFP-Schwann cells, by in vivo microscopy, in the same rodent tibial nerve doxorubicin injury model. Statistical comparison between cohorts in different experimental groups was performed via 2-way analysis of variance (ANOVA) with Tukey’s post-test (blue arrow relationships). Comparison between cohorts of the same experimental group were performed using 1-way ANOVA with Tukey’s post-test (green arrow relationships).

As we were very interested in tracking the biochemical profile of the myelin sheath during regeneration, we analyzed the spectra of Nile Red labeled myelin produced by transplanted, endogenously fluorescent cells over early timepoints of remyelination in three distinct myelin regeneration experiments (Figure 2). Analysis of cell-derived myelin therefore eliminated contamination of our results by analysis of endogenous myelin that may have not been injured by the original demyelination injury.

#### 2.4.1. Fixed Tissue Analysis of Tibial Nerve Remyelination Spectra after Doxorubicin Injury in Rats

Using our established doxorubicin injury/cell transplant method [11], we injected either 5 × 10^5^ eGFP positive syngenic SKP-SCs or SCs into the injured tibial nerves of Lewis rats (no axonal label) nine days after injury with doxorubicin. We used 20 rats in total (10 for each cell group)] with bilateral injury followed by cell therapy, and therefore 40 separate tibial nerves were studied. An individual rat only received one type of cell. On days of harvest, two rats (4 nerves) from each cell group were collected, with the rats being chosen at random. To sample early myelination, we chose our harvest timepoints as post-injury day 21, 24, 27, 30, and 33 based on findings of previous literature [11]. Spectra from myelin regions sampled from BFP positive cells were compared against each other, and against adult myelin of uninjured Lewis rats (12-week-old rodents).

#### 2.4.2. In-Vitro Analysis of Myelination Spectra in Live Culture

We used a co-culture system of DRG explants that pan-express GFP (in both neurons and Schwann cells), and applied either BFP+ve SCs or SKP-SCs to the co-culture system before inducing myelination via addition of ascorbic acid [12]. We designated 3 plates per cell condition for each of the following days post-myelin induction: day 10, day 13, day 16, day 19, and day 22. These days were chosen to represent timepoints when we would likely see myelination, which usually begins in-vitro (at the earliest) 7–10 days after the addition of ascorbic acid [13,14]. The DRG chemotherapy regimen was modified to allow the survival of scant endogenous GFP Schwann cells, such that during co-culture these cells might also proliferate and myelinate, allowing the direct comparison of spectra from supplemented BFP positive cells against those endogenous to the dish.

#### 2.4.3. Intravital Analysis of Tibial Nerve Remyelination after Doxorubicin injury

We employed the above doxorubicin injury model using Thy-1 GFP rats, and performed a unilateral right-sided tibial nerve injury at Day 0 [11]. We then administered either 5 × 10^5^ BFP+ve SKP-SCs or BFP+ve SCs to the injury site at Day 9, for subsequent intra-vital analysis. Our analysis time-points were day 21, 24, 27, 30, 33, and 40 post-injury. We analyzed n = 3 rats for each timepoint.

### 2.5. Surgery

#### 2.5.1. Ethics Statement

All rats were housed independently in the Institutional Animal Research Center for the duration of the experiment, under normal temperature (24 °C) and light cycle (12 h), with unlimited access to food and water. Great care was taken to minimize animal discomfort and provide adequate analgesia in the post-operative period (buprenorphine 0.05 mg/kg twice a day, for three days). All animal interventions were approved by the Institutional Animal Care Committee (ethics protocol # M08124).

#### 2.5.2. Tibial Doxorubicin Injury and Cell Graft Injection

Our methods for tibial nerve doxorubicin injury with cell graft are documented elsewhere [11]. At post-operative day 9, rats received injection of 500,000 (BFP/GFP positive) SKP-SCs or Schwann cells in 5 µL culture medium (DMEM/F12 3:1) into the tibial nerve at the site of doxorubicin injury.

#### 2.5.3. Tibial Nerve Window for Live Imaging

We used adult male Sprague-Dawley Thy-1 GFP rats [15] (colony courtesy of Greg Borschel) to facilitate the intravital imaging experiments, as axons in the PNS of these animals contain endogenous axoplasmic GFP. Animal weights were approximately 200 g at the time of initial doxorubicin injury. The detailed methods of reliably imaging the rodent PNS at high resolution have recently been published elsewhere [10].

### 2.6. Imaging Techniques

#### 2.6.1. Live Rodent Imaging

All live rodent images were captured using a Nikon A1R spectral confocal microscope on a FN1 upright frame using an Apo 25× water dipping objective, NA 1.1, 2 mm working distance, with correction collar.

#### 2.6.2. Imaging of Live Cultures

All cultures were imaged with a Nikon C1si inverted microscope (Nikon, Mississauga, ON, Canada) in combination with a 32 channel spectral detector [Hamamatsu 32 channel multi-anode photomultiplier array, Hamamatsu, Bridgewater, NJ, USA)]. Selected co-culture dishes were given a media change consisting of maintenance media with 10 μM Nile Red, applied for 30 min, followed by a double rinse of maintenance media (5 min each). After this, cultures were bathed in 2 mL of maintenance media, and immediately transferred to the imaging facility.

#### 2.6.3. Imaging of Fixed Tissue

Myelin was analyzed as toroids on axial sections of sciatic nerve. All fixed tissue imaging was performed on the Nikon C1Si inverted spectral confocal as described above.

### 2.7. Cell Culture

#### 2.7.1. Schwann Cell and SKP-SC Primary Culture

Established laboratory protocols were used for the harvest and primary culture of SKP-SCs [16,17,18], as well as neonatal nerve-derived Schwann cells [18,19].

#### 2.7.2. BFP/GFP Transduction of SKP-SCs and SCs

We used established protocols [20] to transduce neonatal SKP-SCs/Schwann cells with lentiviral eGFP and eBFP. Of note, transduced cells were FACS sorted to >95% expression for either BFP/GFP and P75 immunolabeling, which is a robust SC marker for this purpose [21].

#### 2.7.3. DRG Harvest/Culture

Before harvest, 3 cm Fluorodishes (World Precision Instruments, Sarasota, FL, USA) were coated as follows. We combined laminin (4 μg/mL), PDL (20 μg/mL) (both BD Biosciences, San Jose, CA, USA), and 1 mL rat tail collagen Type 1 (Sigma, St. Louis, MO) in 10 mL sterile chilled water, filtered it through a 70 μM filter, and added 500 μL Matrigel (Corning Life Sciences, Corning, NY, USA) to the ice-cold filtrate. Dishes were exposed to this solution for 10 s, with the excess removed by pipette. They were left to dry for 2 h.

DRG explants were obtained from p3 pan-GFP expressing Sprague Dawley (SD) rats (CAG promoter) [22]. Postnatal Day 3 rat pups were sacrificed by decapitation and placed immediately on ice. After exposing the spine a complete rostro-caudal laminectomy was performed, exposing the spinal cord and allowing access to the DRGs. DRGs were harvested with a #5 jeweler’s forceps and stored in chilled Neurobasal media. We harvested 25 DRGs maximum at a single session (1 pup), taking approximately 10 min, in an effort to minimize the degradation of the tissue. DRGs were then trimmed of excess rootlets using a dissection microscope, and plated under the culture hood using 2 mL maintenance media (1 DRG/dish). DRG maintenance media was Neurobasal media, 20% FBS, 1% Pen/Strep, 0.04% Fungizone (all Gibco Biosciences, Carlsbad, CA, USA), and 50 ng/mL Nerve growth factor (Cedarlane, Burlington, ON, Canada). In addition, DRG media initially contained 7 μM cytosine arabinoside (to kill endogenous SCs), and the DRG dishes were left undisturbed in the culture incubator for 4 days. After this time, cultures were inspected daily, and media changed every 2 days. When ample neurites and minimal endogenous Schwann cells were present, usually at day 6, the cytosine arabinoside was removed from the culture condition. DRGs were then washed 3 times over 3 sequential days with chemotoxin-free maintenance media before co-culture was performed.

#### 2.7.4. Co-Culture

Co-cultures were established by seeding either BFP-SKP-SCs or BFP-SCs at a density of 25,000 cells/mL (2 mL/dish) using established techniques [12]. These co-cultures were grown for approximately 6 days in differentiation medium (3:1 DMEM/F12 (Gibco, Carlsbad, CA, USA) with: NGF 1:20000, N2 supplement (Gibco) 1%, Anti-anti (Gibco) 1%, neuregulin 1:400, forskolin 1:5000, and 5% FBS (Gibco). After BFP positive Schwann cells were visualized aligning with axons, media was changed to myelination medium (as above with 0.1% ascorbic acid, and without neuregulin and forskolin, to encourage myelination [23]). Media changes were every 3 days.

### 2.8. Histology

#### 2.8.1. Tissue Processing

All harvested tissue was placed in 10% formalin overnight and then cryoprotected in 30% *w*/*v* sucrose solution for 24 h. Following this, tissue was snap frozen in Tissuetek (Sakura Finetek, Torrance, CA, USA) via immersion in −70 °C isopentane on dry ice. Frozen sections were cut at 14 μM thickness by cryotome (Leica Model CM 1900 3-1, Leica Microsystems, Buffalo Grove, IL, USA).

#### 2.8.2. Dye Preparation

Nile Red (Sigma, St. Louis, MO, USA) was prepared from powder into 1 mM stock concentrations in DMSO. Stock solutions were kept at 4 °C protected from light. Working dilutions (10 µM) were prepared immediately before use by mixing stock concentration with dPBS (Gibco, Carlsbad, CA, USA).

#### 2.8.3. Fixed Tissue Staining

Slides were stained by first rehydrating the samples for 10 min in dPBS, followed by exposure to the appropriate dilution of dye, prepared in a standalone 50 mL glass slide rack. Staining time was 30 min, followed by two sequential 10-min PBS washes. Slides were then tapped dry, coverslipped using Fluorosave (Millipore, Billerica, MA, USA), and placed in the dark pending confocal imaging. All images were acquired within 2 h of staining using a Nikon C1Si spectral confocal microscope (Nikon, Mississauga, ON, Canada).

#### 2.8.4. In-Vitro Immunohistochemistry

Immediately after imaging, culture dishes were rinsed twice with PBS and fixed with 10% Formalin for 20 min. The dishes were stored in dPBS with 0.1% sodium azide, protected from light. For primary antibody staining we rinsed twice with PBS for 5 min, then applied 0.1% Triton in PBS for 5 min, followed by a final PBS rinse before applying 5% bovine serum albumin (Sigma, St. Louis, MO, USA) in PBS for 1 h. The BSA solution was removed and primary antibody solution applied overnight at 4 °C. For secondary antibody staining we rinsed the cultures twice with PBS (5 min), and applied (1:200) secondary antibody in PBS directly for 2 h at room temperature. Cultures were then rinsed twice with PBS before imaging. For neurites we used SMI312 (anti-phosphorylated neurofilament), 1:800 (Abcam, Cambridge, MA, USA)/Alexa 488; for BFP labeled cells we used anti-BFP, 1:200 (Biovision, Milpitas, CA, USA)/Alexa 405; for myelin basic protein we used MBP, 1:200 (Santa Cruz, Dallas, TX, USA)/Alexa 555. We did not re-apply Nile Red for immunohistochemical analysis.

### 2.9. Statistics

Multiple timepoint comparisons of means between replicate measurement populations were performed using a two-way analysis of variance (ANOVA) followed by a Tukey’s post-hoc test, in the following circumstances:

1. Fixed Tissue SKP myelin regions (Day 21, n = 24; Day 24, n = 24; Day 27, n = 24; Day 30, n = 33; Day 33, n = 37; Adult, n = 10) vs. Schwann cell myelin regions (Day 21, n = 14; Day 24, n = 22; Day 27, n = 16; Day 30, n = 19; Day 33, n = 17; Adult, n = 10); independent replicate measurements.
2. In vitro transplanted BFP-SKP-SC myelin regions (Day 13, n = 18; Day 16, n = 15; Day 19, n = 15; Day 22, n = 20.) vs. transplanted BFP-SC myelin regions (Day 13, n = 19; Day 16, n = 15; day 19, n = 29; day 22, n = 30); independent replicate measurements.
3. In vitro endogenous GFP-Schwann cell myelin regions, co-cultured with SKP-SCs (Day 13, n = 19; Day 16, n = 17; Day 19, n = 31; Day 22, n = 20) vs. endogenous GFP-Schwann cell myelin regions, co-cultured with Schwann cells (Day 13, n = 11; Day 16, n = 12; Day 19, n = 30; Day 22, n = 16); independent replicate measurements.
4. In vivo living SKP-SC myelin regions (Day 21, n = 7; Day 24, n = 28; Day 27, n = 5; Day 30, n = 31; Day 33, n = 25; Day 40, n = 15) vs. in vivo living Schwann cell myelin regions (Day 21, n = 8; Day 24 n = 28; Day 27 n = 27; Day 30 n = 20; day 33, n = 15; day 40, n = 17); independent replicate measurements.

Single measurement comparison of means within the above replicate measurement populations was performed using a one-way ANOVA followed by a Tukey’s post-hoc test.

## 3. Results

### 3.1. Spectral Interrogation of Cell Graft-Derived Myelination

#### 3.1.1. Fixed Tissue Analysis

**Figure 3:**
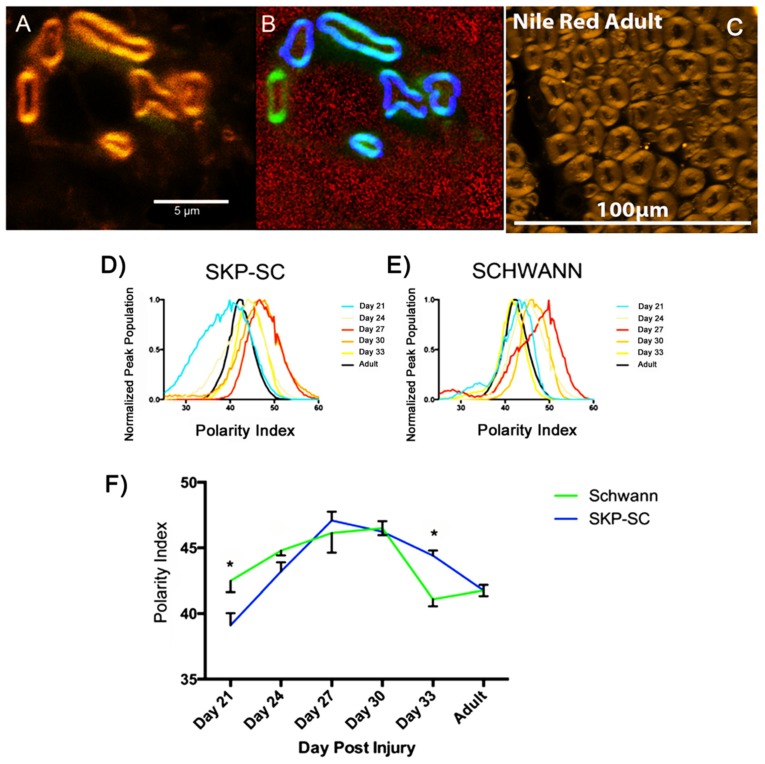
Fixed tissue analysis of SKP-SC and Schwann cell myelination. 24 days after doxorubicin injury and 15 days after cell injection, GFP positive Schwann cells provide myelin to demyelinated axons (not labeled). (**A**) Myelinating GFP positive SCs are visualized (Myelin stained with Nile Red) (**B**) Quantitative pseudocolor image of A (NR stained myelin = green) demonstrating hand-drawn blue selection regions specific to myelin conferred by GFP positive Schwann cells. (**C**) Nile Red stained myelin of adult, non-injured Lewis rats. (**D**) Polarity index distributions from SKP-SC myelin regions. Young myelin (Day 21) exhibited a blue shifted polarity index. The polarity index of myelin increased through Day 24 and 27, before returning toward the polarity index of adult control (uninjured) myelin by Day 33. (**E**) Averaged polarity index curves from Schwann cell myelin selection regions. A similar trend is observed with young myelin progressing from a blue shifted polarity index to a red shifted polarity index by Day 27 and 30, following a return to approximate the polarity index of adult control myelin by day 33. (**F**) Averaged polarity index peak values for myelin regions as outlined in A and B. Day 21 SC PI > SKP-SC PI and Day 33 SKP-SC PI > SC PI (Two-way ANOVA α0.05, Tukey’s post-hoc test), however both distributions maintained a biphasic appearance and there was no statistical difference between the distributions (Two-way ANOVA).

Both SKP-SCs and Schwann cells, injected into the nerve, nine days following doxorubicin, produced new myelin, ensheathing previously denuded axons. This newly regenerated myelin had a polarity index (PI) comparable to that of adult myelin from uninjured nerves (Figure 3, Day 21), a consistent finding independent of the cell type analyzed.

While having a relatively low average Polarity Index young myelin also had a wider and more heterogeneous distribution of measured Polarity Index values. These distributions narrowed after Day 27, with the elimination of secondary spectral peak populations (Schwann cell day 21 and 27) with further narrowing by day 33. Furthermore, there was a trend for the Polarity Index of regenerating myelin to increase over a 6–9 day time period, and to display a maximum at Day 27 or 30 in our model. Following this, there was a return toward the Polarity Index of adult control myelin, of which the Day 33 time-points of both cell groups began to converge.

Within the context of this observed trend, there were time-point specific differences apparent for the Polarity Index of SKP-SC and Schwann cell transplant-derived myelin (Day 21: SKP-SC<SC; Day 33: SKP-SC > SC; 2-way ANOVA (α = 0.05), Tukey’s post-hoc test). There was not a significant difference between the two distributions on the basis of cell type (2-way ANOVA α0.05). However, the “Day Post-Injury” is a highly significant factor (two-way ANOVA, α = 0.05, *p* < 0.0001), and in general demonstrates that the bluest shifted myelin (Day 21, Adult) was significantly different in PI than the most red-shifted myelin (Day 27,30).

#### 3.1.2. In Vitro Analysis of Live Myelin

**Figure 4:**
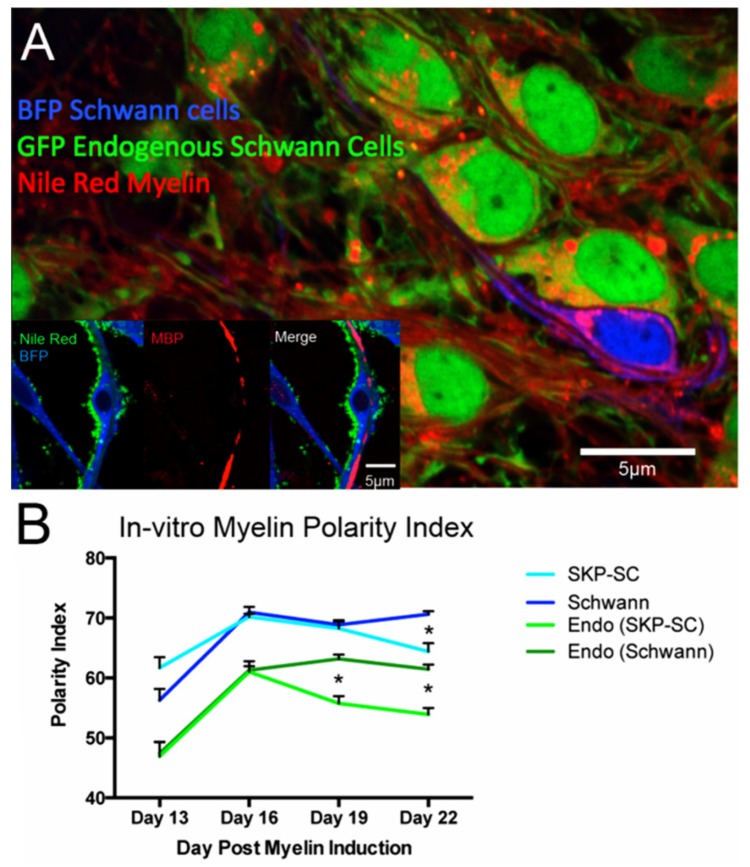
Spectral analysis of an in-vitro co-culture system of GFP expressing DRGs and Schwann cells, co-cultured with either BFP expressing SKP-SCs or Schwann cells. (**A**) BFP (transplanted) Schwann cells and GFP (endogenous) Schwann cells myelinate in live culture after myelination was induced by ascorbic acid (Image at 19 days post-induction of myelination). Inset: Post-fixation, SKP-SCs demonstrate MBP positive myelin (Image at 16 days after myelin induction. 3-channel unmix (blue = BFP, green = Nile red, Red = MBP/Alexa555). Of note, effective tissue processing for MBP staining involves detergent exposure, and hence formation of lipid rich Nile Red positive micelles that accumulate on the SKP-SC border. However, Nile Red stained myelin and MBP still co-localize to the expected location of the myelin sheath. (**B**) Averaged Peak Polarity index of in-vitro myelin selection regions from transplanted BFP+ve Schwann and SKP-SCs, as well as the endogenous Schwann cell populations of those respective conditions (Endo-SKP, Endo-Schwann).

We analyzed the myelin produced by 4 distinct cell groups: (1) Exogenously added SKP-SCs, (2) Exogenously added SCs, (3) Endogenous GFP expressing Schwann cells of the BFP-***SKP***-SC/DRG co-culture [EndoSC(SKP-SC)] and (4) Endogenous GFP expressing Schwann cells in the BFP-***SC***/DRG co-culture [EndoSC(SC)]. Interval comparison of the myelin Polarity Index of all cell groups yielded a similar observed biphasic evolution of Polarity Index over early myelination time points (Figure 4). Of note the Polarity Index of living in vitro myelin was markedly more red-shifted than that of the fixed tissue myelin, though the pattern of emission signature changes over time remained similar.

Robust myelination could be quantified in all cohorts, by endogenous Schwann cells as well as exogenously applied SKP-SC and Schwann cells. Myelin typically began to appear at 13 days after the induction of myelination with ascorbic acid. The experiment was limited in length by the viability of DRGs in the co-culture after 22 days (a total of 40 days total of culture time for the DRGs).

In general, early in-vitro myelination was characterized by a relatively low Polarity Index. This was followed by an abrupt increase at Day 16, and then a gradual decline with increasing age. The myelin spectra from both SKP-SCs and endogenous Schwann cells co-cultured with SKP-SCs achieve this dynamic faster than their Schwann cell co-culture counterparts (Day 19 and 22 Endo(SKP) vs. Endo(SC); Day 22 SKP-SC vs. SC; 1-way ANOVA *p* < 0.05) In addition, the Polarity Index curves of transplanted cells are significantly different from their endogenous counterparts (2-way ANOVA α = 0.05).

For the myelin-specific Polarity Index derived from the BFP cells, there was no significant difference between the curves of these two cell conditions (two-way ANOVA, α = 0.05). Within an individual cell type, the differences in Polarity Index between time points was often very significant, demonstrating that we are observing a real effect with the dynamic changes in myelin spectra [2-way ANOVA, Tukey’s post-hoc test]. The only significant difference between SKP-SC and SC myelin Polarity Index occurred at day 22 (2-way ANOVA *p* < 0.01).

For the myelin-specific Polarity Index derived from endogenous Schwann cells, there was no difference between the curves derived from endogenous SC’s cultured alongside BFP-SKP-SC’s or endogenous Schwann cells cultured alongside BFP-Schwann cells (2-way ANOVA, α = 0.05). Within an individual cell type however there were significant spectral changes with time (2-way ANOVA, Tukey’s post-test). Interestingly, there was a significant difference between the spectral Polarity Index of myelin derived from exogenous cells vs. that of endogenous Schwann cell myelin in any group. (SKP-SC > endoSKP-SC, Schwann > endoSchwann; 2-way ANOVA, α = 0.05).

#### 3.1.3. Intravital Analysis

**Figure 5:**
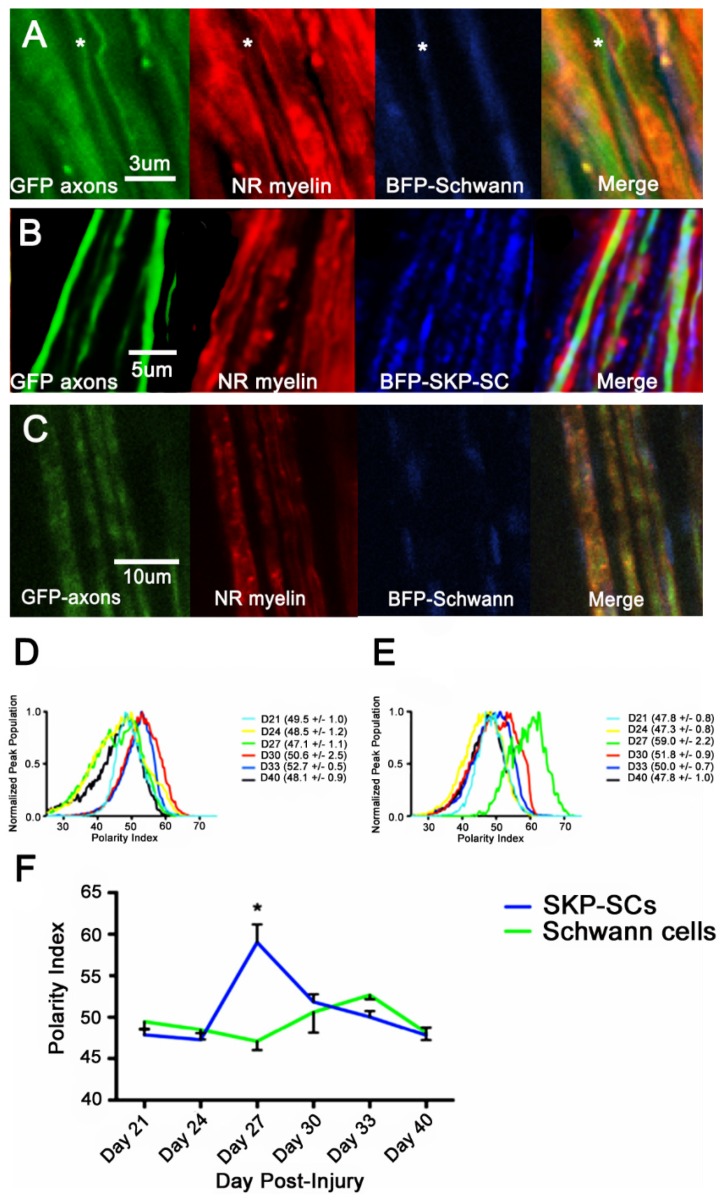
Intravital polarity index analysis of SKP-SCs and Schwann cell transplants over sequential days of myelination. **(A**–**C**) High magnification intravital images of BFP+ve SKP-SC and Schwann cell myelination, demonstrated as three-channel images. Green = GFP+axons, Red = Nile Red stained myelin, Blue = BFP+ve cytoplasm and nucleoplasm of injected cells. (**A**) 21 days post-doxorubicin injury, 11 days post BFP-Schwann cell injection. * = Thinly myelinated axon (**B**) 27 days post-doxorubicin injury, 19 days post BFP-SKP-SC injection. (**C**) 40 days post doxorubicin injury, 31 days post BFP-Schwann cell injection. (**D**) BFP-Schwann cell myelin PI average distributions (**E**) BFP-SKP-SC myelin PI average curves. F) Peak Polarity Index values vs. time, Schwann cells (green line) and SKP-SC cells (blue line). SKP-SC day 27 myelin is red shifted compared to Day 27 SC myelin (Two-way ANOVA, α = 0.05, Tukey’s post-hoc test). Both data demonstrate a significantly changing biphasic polarity index with time, moving from an early blue shifted Polarity Index, through a red shifted Polarity Index, and back to a blue shifted Polarity Index at the most mature time points.

Intravital analysis of cell transplant derived myelin demonstrated for a third time that myelin membrane biochemistry follows a progression from low to high polarity, and back again over the first two weeks of observable remyelination (Figure 5). In general, there was no significant difference between the SKP-SC and SC distributions (2-way ANOVA, α = 0.05)

SKP-SC myelin Polarity Index underwent a statistically significant change over the course of remyelination (Day 21,24 < Day 27 > Day 30,40) in our doxorubicin injury model (2-way ANOVA, α = 0.05). Transplanted Schwann cell myelin also displayed a similar pattern (Day 27 SC < Day 33). Unlike the previous models, the SKP-SC derived myelin developed in a manner that allowed an earlier (or potentially more coordinated) progression through the “polar” phases, as compared to the Schwann cell derived myelin. There was a significant difference between SKP-SC myelin Polarity Index and Schwann myelin Polarity Index at Day 27 (2-way ANOVA, *p* < 0.001).

## 4. Discussion

### 4.1. Environmentally Sensitive and Solvatochromic Membrane Dyes

A rapidly expanding field biophysical science is the analysis of the composition of membranes by environmentally sensitive probes [1]. These probes are fluorescent molecules that change their fluorescent characteristics (absorption/emission maxima, fluorescence intensity/duration) depending on their molecular micro-environment [1]. Changes in the state of the membrane (liquid vs. liquid ordered vs. gel), as well as changes in membrane composition/polarity, are environmentally specific events that can translate into fluorescence dynamics; the specific changes observed also depend on the type of dye employed.

Certain classes of environmentally sensitive fluorophores obtain very large dipoles when excited by the appropriate wavelength of light, as compared to their base, non-excited state [1]. When this occurs, the surrounding solvent sphere molecules may respond with the phenomena of “solvent relaxation”, aligning their dipoles to stabilize the new dipole of the fluorophore. The degree of solvent relaxation determines the final energy of the excited emission state of the fluorophore, as the relevant solvent relaxation occurs faster than emission. The greater the stability of the excited state generated by solvent relaxation, the lower the energy (longer wavelength) of the emitted photon. This phenomenon is termed solvatochromatism.

### 4.2. Regenerating Myelin Displays a Biphasic Progression in Polarity with Increasing Maturity

Our overall goal was to develop a spectral based imaging technique that could be used to interrogate the regenerative maturity of peripheral nerve myelin in live imaging scenarios. Using this novel technique, the primary finding of these experiments is that Nile Red, a solvatochromic fluorophore that to date has been primarily used to analyze the biochemical properties of experimental membranes, can be utilized as an indicator of the biochemical maturity of regenerating myelin in the peripheral nervous system. We demonstrate using both in-vitro and in-vivo models that the myelination conferred by SKP-SC and Schwann cells follows a biphasic evolution of its membrane polarity over time. We suggest that blue shifts in Nile Red polarity index may be due to increases in lipid order and lateral lipid packing, thereby representing an increased fraction of myelin membrane cholesterol as per Kucherak et al. [24].

**Figure 6:**
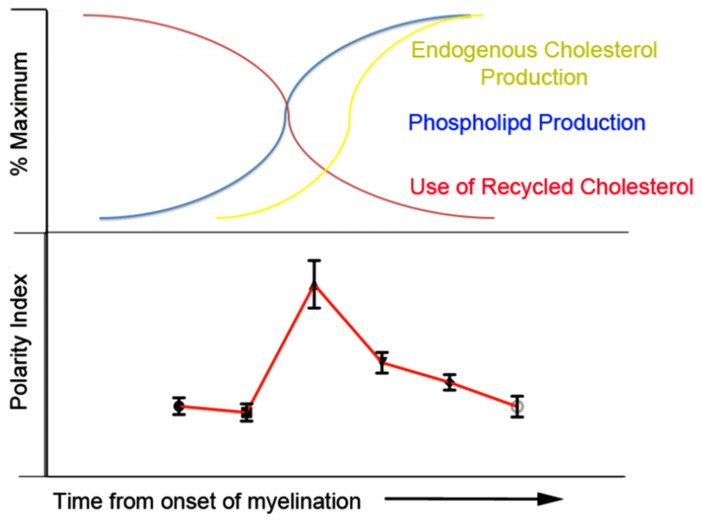
A theory regarding the biphasic evolution of myelin polarity index over early time-points of remyelination. Initially Schwann cells extensively incorporate salvaged cholesterol, generating new myelin with a low polarity (more lipophilic character). Upregulation of phospholipid production occurs during a nadir of cholesterol levels, while the cell is switching from exogenous cholesterol use to endogenous cholesterol production. The dominant addition of phospholipid to the myelin during this time results in a transient increase in the Polarity Index of myelin signatures. Endogenous cholesterol production then reaches full capacity and is gradually incorporated into the phospholipid rich myelin, resulting in a gradual return of myelin Polarity Index to lower, mature levels. These processes occur over approximately two weeks after injury [25,26,27,28,29,30,31].

The biochemistry of remyelination has been well studied in a group of seminal papers that used radiolabelling techniques to determine the proportional contributions of lipid de-novo synthesis, as compared to lipid recycling during regeneration of the injured PNS [25,26,27,28,29,30,31]. Although these studies were initially performed after axonotmetic injury, the findings were later validated in a model of primary demyelination (tellurium) [31]. Fascinatingly, cholesterol of degenerated myelin is almost entirely conserved, and incorporated into the new myelin membrane from the outset of remeylination [32]. Consistent with this, actual de-novo cholesterol synthesis is down-regulated [27] in Schwann cells in early Wallerian degeneration. The recycled cholesterol is formed into cholesterol esters by macrophages, whereby it associates with Apolipoprotein E [28] to become available for transport, and thereby interaction with LDL receptors inherent on Schwann cells during PNS regeneration [33]. Endogenous cholesterol production does return however [31], likely due to low levels of available lipoprotein associated cholesterol stimulating the function of HMG-CoA reductase [34]. Congruently, synthesis of new myelin phospholipid begin 5 days after Tellurium injury, and reaches a peak level plateau when intracellular cholesterol levels may be at this proposed nadir [31]. In a simplified way, if the cholesterol to pure phospholipid ratio in a membrane system represents the degree of membrane lipid order, we can see that this ratio may be in flux during early remyelination. We therefore propose that the interplay between cholesterol reuse, phospholipid production, and endogenous cholesterol production forms the basis of spectral changes that we observed with progressive myelin maturity (Figure 6).

### 4.3. Composition of Culture Media May Influence Myelin Biochemistry

It may be that the close association of lipid and cholesterol rich cells (macrophages/fibroblasts) present within the DRG are able to confer some of their metabolites to endogenous Schwann cells before they begin to myelinate, making their myelin on average less polar than that of transplanted, non-serum raised cells. Schwann cells are well known to express LDL receptors, and receive lipid raw material from adjacent cells via an ApoE mediated mechanism [28,35,36]. Our in-vivo images often demonstrated extensive lipid rich deposits in Schwann cells, particularly in GFP positive endogenous cells, and particularly during early myelination time-points (data not shown).

### 4.4. Myelination by Schwann Cells and SKP-SCs Proceeds in a Biochemically Similar Fashion

Interestingly, the condition- specific biphasic polarity index of remyelination is similar between SKP-SCs and Schwann cells, suggesting that myelin derived from SKP-SCs is similar to that derived from Schwann cells on a fundamental, biochemical level. Previous work from our lab and others has made efforts to demonstrate the ability of SKP-SCs to behave as Schwann cells over increasingly detailed levels of biological scrutiny [11,16,18,23]. We compellingly demonstrate here that SKP-SCs have a SC phenotype on a very fundamental level, as both cells seem to have similar machinery driving the content and timing of new myelin formation in the PNS.

## 5. Conclusions

We have demonstrated that Nile Red emission spectra changes in a biphasic pattern when used to probe remyelination chemistry during the first two weeks of myelination, a finding that was consistent in each experimental paradigm we tested. The changes in polarity index demonstrated are plausible, in the context of what is known about the chemistry of early remyelination in the PNS following injury. We have also demonstrated the similarity of Schwann cell and SKP-SC derived myelin on a biochemical level, as these cells progress through the early stages of remyelination in a tibial nerve doxorubicin injury model.

The ability of Nile Red to indicate biochemical “phase” of early myelin regeneration is a unique and powerful tool for the high resolution, high magnification analysis of regenerative phenomena, in vitro as well as in-vivo. Future directions include the detailed investigation of myelin spectra by correlation with high resolution mass spectroscopy techniques [37] in non-living tissue, to enable further commentary regarding the biochemical changes underpinning our observed findings. Potential incorporation of solvatochromic analysis into gold-standard measures of myelin thickness assessment (morphometry) may allow a faster, more accurate assessment of the true regenerative nerve environment. Our data also raise new questions; for example, it is suggested from our in-vitro studies that the culture constituents present before initiation of myelination co-culture may affect the overall biochemistry of the myelin produced by cells raised in these different environments. While solvatochromic analysis suggests this phenomenon, it also provides a novel means to further study this experimental question in living cell cultures. Going forward, spectral analysis of solvatochromic myelin-incorporated dyes may become a valuable methodology for examining myelin biochemistry in a non-invasive fashion, ultimately making it applicable for the study of PNS myelin, CNS myelin, live animals and potentially humans.

## Abbreviations

ANOVA	Analysis of Variance
bFGF	Beta fibroblast growth factor
BFP	Blue fluorescent protein
BSA	Bovine serum albumin
CMV	Cytomegalovirus
CNS	Central nervous system
CW	Continuous wavelength
DLPC	Dilauroylphosphatidylcholine
DMEM	Dubelco’s modified eagle media
DMSO	Dimethylsulfoxide
DNA	Deoxyribonucleic acid
DRG	Dorsal root ganglia
ECM	Extracellular matrix
EGF	Epidermal growth factor
eGFP	Enhanced green fluorescent protein
ER	Endoplasmic reticulum
ESMD	Environmentally sensitive membrane dyes
F12	Ham’s F-12 Nutrient Mixture
G	Gravity
GFP	Green fluorescent protein
HEK	Human embryonic kidney
HMG-CoA	3-hydroxy-3-methylglutaryl-coenzyme
LV	Lentivirus
MBP	Myelin basic protein
mRNA	Messenger ribonucleic acid
NA	Numerical aperture
NF	Neurofilament
NGF	Nerve growth factor
NRSSA	Nile red solvatochromic shift assay
OD	Overdose
p# (eg. P3)	Post natal day # (eg. Post natal day 3)
P0	Myelin protein zero
PBS	Phosphate buffered saline
PDL	Poly-d-lysine
Pen/Strep	Penicillin and streptomycin
PI	Polarity index
PLP	Proteolipid protein
PMP22	Peripheral myelin protein 22
PNS	Peripheral nervous system
SCAP	SREBP cleavage activation protein
Shi	Shiverer (gene)
SKPs	Skin-derived precursor cells
SKP-SCs	Skin-derived precursor Schwann cells
SREBP	Sterol regulatory element binding protein
VSV-G	Vesicular stomatitis virus glycoprotein
